# Ovarian hyperstimulation syndrome in a spontaneous pregnancy with invasive mole: report of a case

**DOI:** 10.4314/pamj.v9i1.71198

**Published:** 2011-06-27

**Authors:** Myriam Rachad, Hikmat Chaara, Fatim Zahra Fdili, Hakima Bouguern, Abdilah Melhouf

**Affiliations:** 1 Department of Obstetrics and Gynecology II, CHU Hassan II, Fez, Morocco

**Keywords:** Ovaries, hyperstimulation syndrome, spontaneous pregnancy, invasive mole

## Abstract

It is known that most cases of Ovarian Hyperstimulation Syndrome (OHSS) are associated with the therapies for ovulation induction. However, OHSS may rarely be associated with a spontaneous ovulatory cycle, usually in the case of multiple gestations, hypothyroidism or polycystic ovary syndrome. We report a case of severe OHSS in spontaneous pregnancy with invasive mole in a 34 years old woman. The clinical picture showed abdominal pain, massive ascites, nausea, dyspnea and amenorrhea. After imaging examinations and laboratory tests, the diagnosis was established. The patient was managed expectantly with no complications. Although spontaneous ovarian hyperstimulation is a rare entity, it is important that the physician recognizes this condition. Prompt diagnosis and successful management is likely to avoid serious complications, which may develop rapidly.

## Introduction

Ovarian Hyperstimulation Syndrome (OHSS) is a rare, iatrogenic complication of ovarian stimulation with follicule stimulating hormone (FSH) medications. OHSS was first described in 1943, and the first fatal cases were documented in 1951 [1]. Severe OHSS is estimated to occur in approximately 1% of all gonadotropin cycles [1]. A study done in 2005 using data from the Finnish registry reported an incidence of severe OHSS of 1.4% per cycle, with an individual risk per patient of 2.3% over a mean number of 3.3 cycles [2]. In spontaneous pregnancy, OHSS is an extremely rare event. Here we present a severe OHSS case, in a spontaneous pregnancy with invasive mole.

## Patient and case report

The patient, a 34 years old woman, gravida 3 para 3, was referred to our department on a suspicion of malignant ovarian disease. The patient had no significant past medical or surgical history. She presented a month before with abdominal distension, pain, nausea and dyspnea. Her obstetrical history revealed three normal term deliveries (5, 3 and 1 years before); her menarche occurred at the age of 13 and subsequent menses were regular. Her last menstrual period occurred 12 weeks prior to admission. She had no history of ovulation induction or any medication within the last 6 months. Family history was negative for both polycystic ovary syndrome and hydatiform mole.

The physical examination revealed normal vital signs and a severely distended abdomen with evidence of ascites but without palpable masses. Vaginal examination revealed a gravid cervix but the evaluation of the uterus was difficult. Laboratory tests revealed normal concentration of heamoglobin, haematocrit, blood electrolytes, creatinine and blood urea nitrogen, and coagulation profile. Beta human chorionic gonadotrophin was >2 000 000 mui/ml.

**Figure 1:**
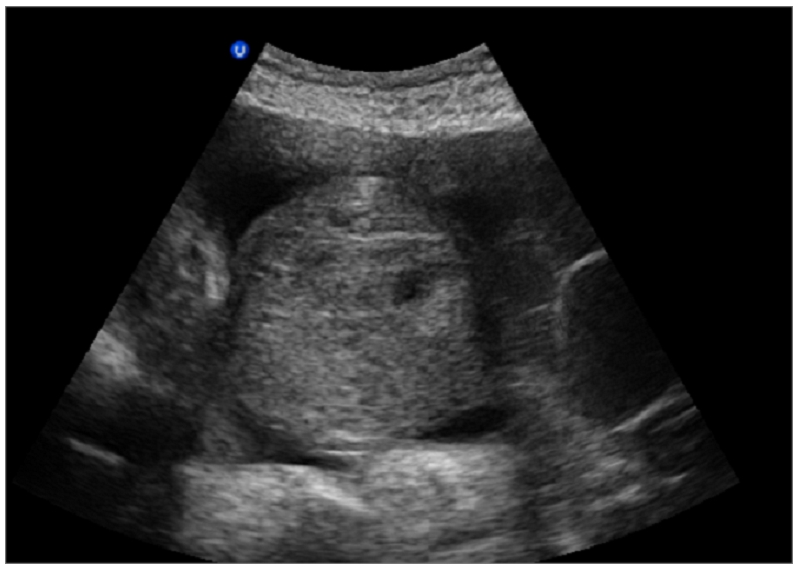
abdominal ultrasonography showing a heterogeneous mass in the uterine cavity that was difficult to distinguish from the myometrium in a patient with Ovarian hyperstimulation syndrome in a spontaneous pregnancy with invasive mole

**Figure 2:**
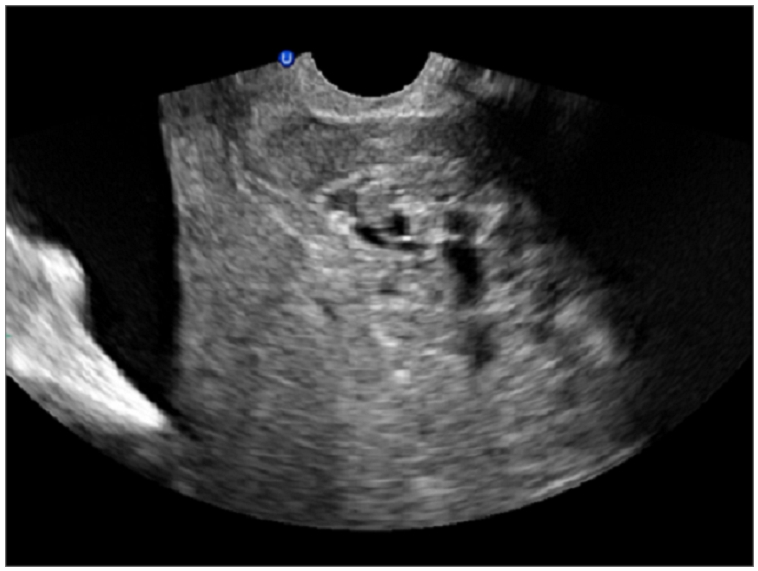
transvaginal ultrasonography showing a heterogeneous mass in the uterine cavity in a patient with Ovarian hyperstimulation syndrome in a spontaneous pregnancy with invasive mole

**Figure 3:**
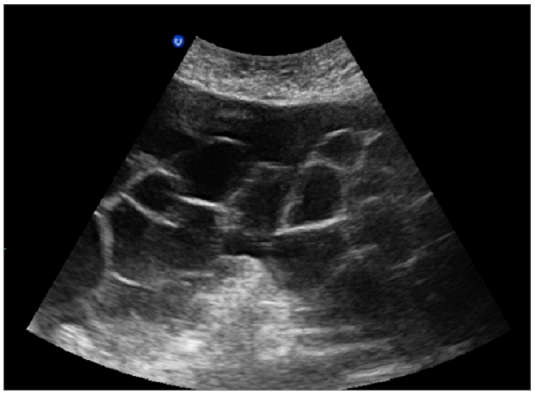
bilateral multilocular cystic masses in a patient with Ovarian hyperstimulation syndrome in a spontaneous pregnancy with invasive mole

**Figure 4:**
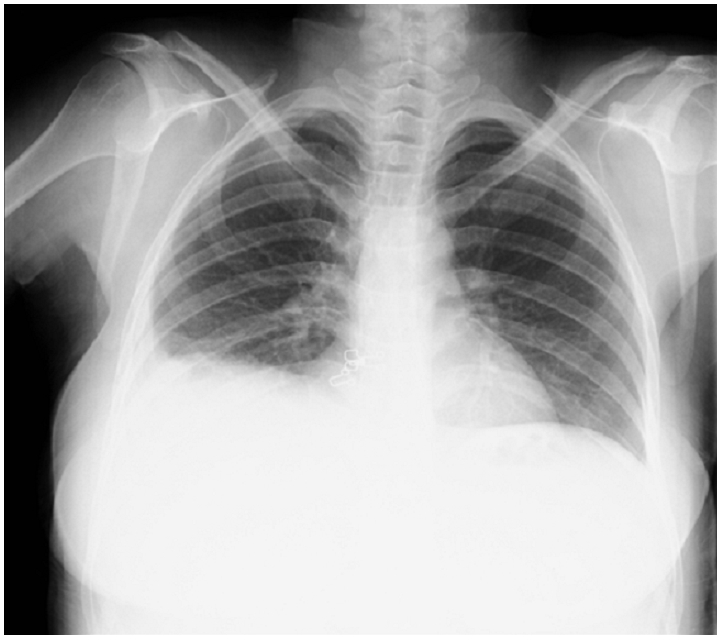
chest X-ray showing right pleural effusion in a patient with ovarian hyperstimulation syndrome in a spontaneous pregnancy with invasive mole

**Figure 5:**
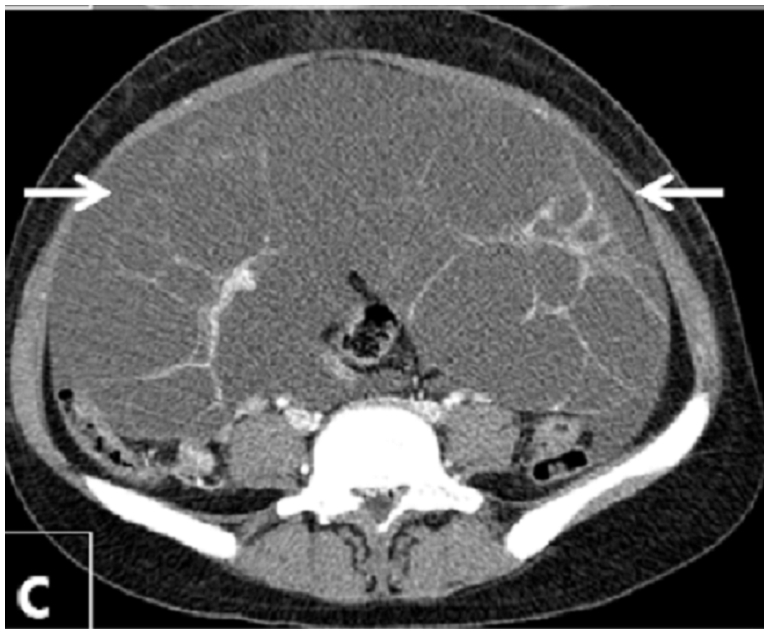
Abdominal pelvic scan showing enlarged multicystic ovaries (16cm rights, 12cm lefts) with ascites in a patient with ovarian hyperstimulation syndrome in a spontaneous pregnancy with invasive mole

Abdominal and transvaginal ultrasonography showed a heterogeneous mass in the uterine cavity that was difficult to distinguish from the myometrium (Figure 1, 2), with bilateral multilocular cystic masses (Figure 3) and a large amount of ascites. Right pleural effusion was also present on chest X-ray chest (Figure 4). Abdominal pelvic scan showed enlarged multicystic ovaries (16cm rights, 12cm lefts) with ascites (Figure 5).

A gestational trophoblastic tumor was expected, and was confirmed by Doppler ultrasonography and MR imaging which showed deep myometrial invasion. A diagnosis of severe OHSS, with gestational trophoblastic tumor was made.

Immediately after admission, infusion therapy was started, consisting of normal saline-infusion 0.9% 1000 ml, glucose 5% 1000 ml, four times 50 ml of human serum albumin 20%, and low molecular weight heparin 5000 IU 2 × 1 per day.

Following thoraco-abdomino-pelvic scan and brain MR imaging, the patient was scored as high risk and chemotherapy was initiated with etopside, methotrexate, and cyclophosphamide.

**Figure 6:**
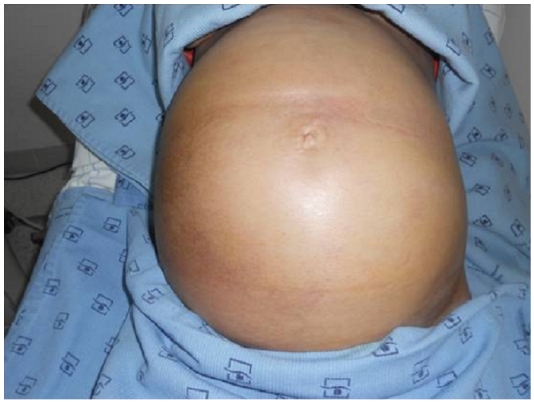
significant increase in abdominal girth in a patient with ovarian hyperstimulation syndrome in a spontaneous pregnancy with invasive mole

Body weight, abdominal circumference, intake and outputs, ultrasonography, and laboratory studies were monitored strictly. Renal function was not disturbed and there were no serious changes in serum electrolytes. The patient's girth increased starting from the 6^th^ day (Figure 6) requiring abdominal paracentesis for the drainage of the massive ascites necessary; 2000 ml of ascitic fluid was removed.

Because the patient has 3 children, and to decreases the number of cures necessary to obtain complete remission of the disease, hysterectomy was favored.

**Figure 7:**
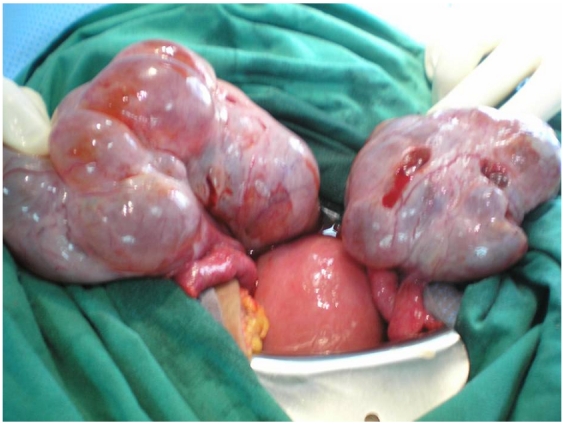
laparotomy of the patient with ovarian hyperstimulation syndrome in a spontaneous pregnancy with invasive mole. The uterus was of 12 weeks gestation in size, and the ovaries were multilobulated, each measuring 12 cm in diameter, and containing cystic structures

On laparotomy, 3000 ml ascitic fluid was removed. The uterus was of 12 weeks gestation in size, and the ovaries were multilobulated, each measuring 12 cm in diameter, and containing cystic structures (Figure 7). An hysterectomy and ovarian drilling were performed. Pathological examination of the uterus confirmed the invasive mole.

The clinical conditions were stable during the following weeks; BHCG was negative after a second round of chemotherapy. Ultrasound performed two months after surgery showed ovaries of normal dimensions and structure.

## Discussion

Ovarian hyperstimulation syndrome in spontaneous pregnancy is an extremely rare event. Under certain circumstances such as twin pregnancies, the possibility of its existence may be higher because of higher HCG concentrations during the early pregnancy [3].

OHSS has life threatening complications such as venous and arterial thromboembolism. Indeed, Schenker and Ezra reported the death of patients with OHSS to be caused by these complications [4]. Consequently, one must be aware of the rare but possible occurrence of OHSS in spontaneous pregnancy, in order to prevent its complications.

The pregnancy pathology with the highest HCG values is the mole pregnancy. However, even within this entity, the prevalence of OHSS is low [3]; only few cases have been published [3, 5–7]. The clinical pictures were associated with ascites and at least one of these cases showed a severe complication by disseminated intravascular coagulation and renal failure [5]. To our knowledge, the case we presented is a rare example of spontaneous OHSS and one of the very few examples of OHSS with invasive mole.

Spontaneous forms of OHSS generally develop between 8 and 14 weeks of amenorrhea, differing from iatrogenic OHSS, which usually starts between 3 and 5 weeks of amenorrhea. The recent identification of mutations in the follicle stimulating hormone (FSH) receptor gene, which display an increased sensitivity to hCG and are responsible for the development of spontaneous OHSS, helps us to understand this problem [8]. In iatrogenic OHSS, the follicular recruitment and enlargement occur during the administration of exogenous FSH. In the spontaneous form however, the follicular recruitment and growth occur later through the promiscuous stimulation, by pregnancy-derived hCG, of a mutated FSH receptor that is abnormally sensitive to hCG or a wild type FSH receptor in the presence of abnormally high levels of hCG. Thus, the symptomatology of spontaneous cases of OHSS usually develops at 8 weeks’ amenorrhea and culminates at the end of the first trimester of pregnancy [9].

In the literature, different cases were reported in which spontaneous pregnancy with OHSS and hypothyroidism was found together. It was claimed that high levels of thyroid stimulating hormone can stimulate ovaries in women with hypothyroidism and can cause ovarian hyperstimulation [10, 11]. In our case, hypothyroidism was not present.

Hyperstimulated ovaries release a number of vasoactive mediators under the influence of hCG. These include vascular endothelial growth factor (VEGF) and several pro-inflammatory cytokines that interact to produce the characteristic pathophysiology of OHSS. This is marked by increased capillary, permeability, leakage, of fluid from the vasculature, third space fluid accumulation and intravascular dehydration [1].

Different classification systems for OHSS have been proposed, which generally identify a mild, moderate, and severe subtype with varying internal grades of severity. In order to simplify the classification of OHSS, we used a classification system adapted from that proposed by the Royal College of Obstetricians and Gynaecologists in 2006 [12, 13]. We identify mild OHSS, in which patients have abdominal bloating and mild abdominal pain; moderate OHSS, characterized by nausea, vomiting, moderate abdominal pain, and ultrasound evidence of ascites; severe OHSS, identifiable by clinical ascites, oliguria, hematocrit > 45%, and hypoproteinemia; and critical OHSS, with tense ascites, oliguria or anuria, hematocrit > 55%, and white blood count > 25,000.

The management of OHSS is tailored to the degree of severity. Early recognition and prompt appropriate treatment will avoid serious sequelae. Severe OHSS requires hospital admission and prompt management to replace lost intravascular volume and prevent its potentially fatal complications namely renal failure and thromboembolic events [14]. These patients should be closely monitored to ensure they do not progress into the critical category. In patients with significant ascites, paracentesis is helpful by decreasing intra-abdominal pressure and improving renal blood flow with a subsequent increased production of urine [15, 16]. Drainage of ascitic fluid in OHSS may be carried out abdominally or vaginally. The vaginal route has the benefit of easier access and avoidance of ovarian trauma [17]. Pleural effusions are not uncommon in OHSS. Thoracocentesis may be necessary to avoid respiratory distress, accompanied by paracentesis in order to prevent fluid from leaking back into the pleural cavity [18].

Medical treatment, undertaken in first line, may be insufficient. In these cases, invasive treatment, using surgical techniques, becomes necessary. Wedge resection of approximately one-third of the ovaries has been reported in two patients who had failed to respond to albumin infusion and paracentesis [19]. In each case a recovery in the ascites, urine output and biochemical parameter was observed. However, surgical intervention should be regarded as a last resort and only considered in consultation with senior clinicians familiar with managing OHSS.

## Conclusion

OHSS is very rare but potentially serious complications on gestational trophoblastic disease. The exact etiology is still unknown although there is much ongoing research. It seems likely that a culmination of factors: higher HCG concentrations during the early pregnancy and mutations in the follicule stimulating hormone (FSH) receptor gene, which display an increased sensitivity to hCG, play a role. It is vitally important to diagnose and manage spontaneous OHSS promptly to prevent any severe complication.
